# Functional interrogation of contextually correct *MYH7* variants using CRaTER-flox gene editing and contractility profiling

**DOI:** 10.1172/JCI192057

**Published:** 2025-11-25

**Authors:** Alexander M. Loiben, Wei-Ming Chien, Ashley McKinstry, Dania Ahmed, Matthew C. Childers, Michael Regnier, Charles E. Murry, Kai-Chun Yang

**Affiliations:** 1 Department of Medicine/Cardiology and; 2 Department of Bioengineering, University of Washington, Seattle, Washington, USA.; 3 Department of Stem Cell Biology and Regenerative Medicine, University of Southern California, Los Angeles, California, USA.; 4 Cardiology/Hospital Specialty Medicine, VA Puget Sound Healthcare System, Seattle, Washington, USA.

**Keywords:** Cardiology, Genetics, Cardiovascular disease, Genetic diseases, Heart failure

**To the Editor:** A major limitation of genetic testing is the frequent reporting of variants of uncertain significance (VUS). Pathogenic variants in myosin heavy chain 7 (*MYH7*) cause hypertrophic cardiomyopathy (HCM) or dilated cardiomyopathy (DCM). While accurate variant interpretation can assist with diagnosis and screening of at-risk relatives, approximately 85% of *MYH7* missense variants are VUS. Here, we describe a functional assay that predicts *MYH7* variant effects in the context of a human sarcomere (1). Assays measuring cell size and contractility (2) have been reported but not rigorously tested with a panel of *MYH7* benign and pathogenic variants to determine their accuracy and utility for variant classification.

While human induced pluripotent stem cells (hiPSCs) and their derivatives can determine variant effects in physiologically relevant cells, a major challenge is the generation of sufficient variant hiPSC lines to establish ranges for normal and disease phenotypes. To address this, we adapted the gene-editing strategy CRISPRa On-Target Editing Retrieval (CRaTER) (3) by combining it with the Cre-lox system, hereafter referred to as CRaTER-flox, to efficiently introduce variants nearly scarlessly into hiPSCs. Using WTC11 hiPSCs, we replaced *MYH7* intron 11 through intron 14 genomic DNA, a pathogenic variant hotspot, on one allele with a repair template using standard CRISPR/Cas9 gene editing (Figure 1A). The repair template contained a loxP-flanked region expressing EGFP followed by *MYH7* intron 11 through intron 14 genomic DNA with a single-nucleotide variant (SNV) of interest. Correctly edited hiPSCs were CRaTER-enriched by transiently activating *MYH7* and flow sorting for EGFP+ hiPSCs (Supplemental Figure 1, A–C; supplemental material available online with this article; https://doi.org/10.1172/JCI192057DS1) (3). Cre recombination removed the floxed region, leaving the desired heterozygous SNV and a 40-nucleotide loxP intronic scar. We generated 2 benign editing control lines (with loxP in intron 11 or 14), 2 benign variant lines (T441M and R453R), 4 pathogenic/likely pathogenic (P/LP) DCM-associated or LV systolic dysfunction–associated (LVSD-associated) variant lines (R369Q, P402L, Q451K, and I457M) (4), and 6 P/LP HCM-associated variant lines (R403L, R403W, R442L, R453C, R453H, and I457T) (Figure 1, B and C). Remarkably, 76.8% of these hiPSC colonies were correctly gene edited nearly scarlessly with CRaTER-flox (Figure 1D and Supplemental Figure 1D), improving upon the prior CRaTER approach (Supplemental Figure 1E) (3).

We first assessed cell size, as estimated by forward scattering area (FSC-A) with flow cytometry as a pathogenicity marker. We measured FSC-A of hiPSC-derived cardiomyocytes (hiPSC-CMs) and calculated z scores based on mean FSC-A values across all benign lines (Supplemental Figure 2A). While hiPSC-CMs expressing DCM or HCM variants were larger compared with hiPSC-CMs expressing benign variants, neither was statistically significant (Supplemental Figure 2B), indicating that FSC-A was unable to reliably discriminate pathogenic from benign variants.

Next, we assessed whether contractile function measured by traction force microscopy (TFM) (5) can accurately determine variant effect. hiPSC-CMs were matured 7 days on micropatterned hydrogels with physiological stiffness to promote adult-like morphology. Paced single-cell twitch force and contractile kinetics were derived from the displacement of fluorescent beads embedded in the hydrogel (Supplemental Figure 2, C and D) (5). Maximum twitch forces were converted to a normal distribution with a logarithmic transformation (Supplemental Figure 2, E and F), and z scores were calculated based on the distribution of benign line means. All benign variants mean force *z* scores were within the normal range (±2 *z* scores), while all pathogenic variants were outside the normal range, demonstrating this assay’s ability to discriminate pathogenic from benign variants (Figure 1, E and F). Furthermore, all HCM-associated pathogenic variants were hypercontractile with faster contraction and relaxation velocity, while all DCM- and LVSD-associated pathogenic variants were hypocontractile with a trend toward slower contraction and relaxation velocity (Figure 1, G–I), suggesting TFM may predict variant-specific cardiomyopathy.

We used the TFM assay to determine the effect of 4 MYH7 VUS (Supplemental Figure 3A). The hiPSC-CMs expressing R434K had normal contractile function, while hiPSC-CMs expressing R403P, R442H, or R442P had reduced contractile function, suggesting the latter 3 may be pathogenic. The functionally abnormal variants are in the blocked head/tail (R442) and blocked head/free head (R403) interfaces, which may affect the stability of the *MYH7* interacting-heads motif (Supplemental Figure 3B). The R434K VUS with a conservative side-chain substitution is located away from the interacting interfaces.

Next, we used CRaTER-flox to introduce variants in exon 18 of *MYH7* (Supplemental Figure 4, A–C) and exon 11 of troponin T (*TNNT2*) (Supplemental Figure 4, D–F), a cardiomyopathy-associated gene with many splicing isoforms, with similar editing efficiency (Figure 1D and Supplemental Figure 4, G and H). The TFM assay accurately discriminated pathogenic from benign variants in these exons. The HCM-associated variants were hypercontractile, while the DCM- and LVSD-associated variants were hypocontractile (Figure 1F), mirroring the results of the *MYH7* exon 12–14 variants. Across all *MYH7* variants studied, all 6 benign variants were in the normal range, while all 13 pathogenic variants were outside the normal range, demonstrating the robustness of this assay in discriminating benign from pathogenic variants. In addition to successfully editing other genomic loci, this near-scarless gene-editing strategy enables the study of genes with multiple splicing isoforms.

Overall, we applied CRaTER-flox, a method that efficiently introduces variants nearly scarlessly into hiPSCs, to enable the functional interrogation of variants in physiologically relevant cell types with contextually correct genetic backgrounds. We interrogated the functional consequences of a panel of benign and pathogenic *MYH7* variants with a cardiomyocyte contractility assay to assess *MYH7* variant effect. This assay could be adapted and validated to clinically classify *MYH7* variants.

## Supplementary Material

Supplemental data

Unedited blot and gel images

Supporting data values

## Figures and Tables

**Figure 1:**
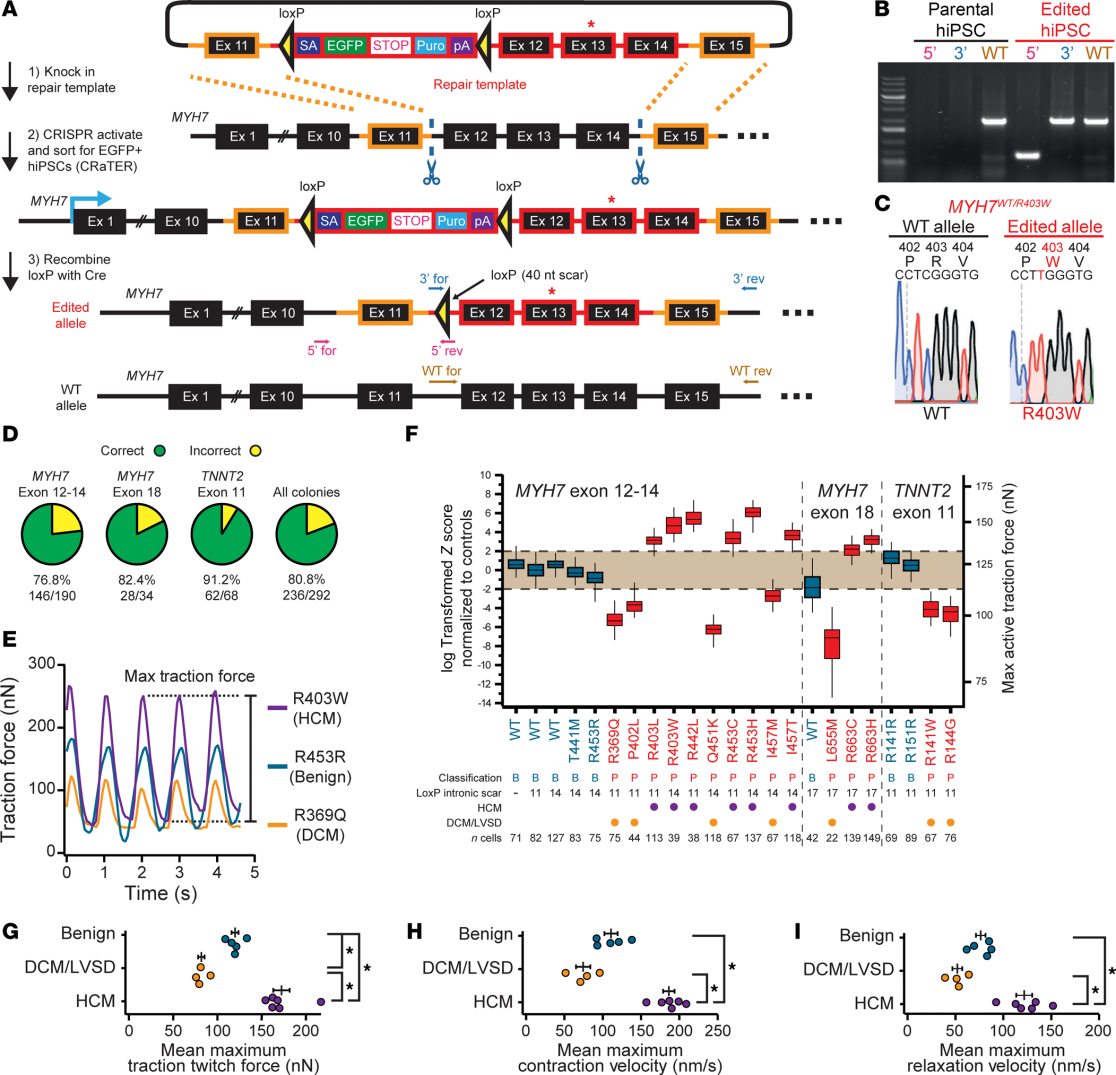
CRaTER-flox efficiently gene edits variants nearly scarlessly into hiPSCs to enable accurate assessment of variant effect. (**A**) CRaTER-flox gene-editing approach. Red asterisk indicates variant. (**B**) Representative genotyping gel using primers indicated in **A**. Parental: WTC11 *MYH7^WT/WT^*. Edited: WTC11 *MYH7^WT/R403W^*. (**C**) Representative sequencing chromatograms. (**D**) CRaTER-flox editing efficiency to generate variant hiPSC lines. (**E**) Representative hiPSC-CM force curves as measured with traction force microscopy. (**F**) Log-transformed maximum hiPSC-CM active traction force *z* scores normalized to benign lines and raw force values. Blue: benign/likely benign (B); red: pathogenic/likely pathogenic (P) variant. Box bounds: upper and lower quartiles; midline: median; whiskers: 1.5 × IQR. Circles: variant-associated cardiomyopathy reported in ClinVar or de Frutos et al. (4). (**G**–**I**) Mean maximum *MYH7* exon 12–14 hiPSC-CM active traction forces (**G**), maximum traction velocity (**H**), and maximum relaxation velocity (**I**). Kruskal-Wallis test, *P* < 0.05; post hoc 2-tailed Mann-Whitney *U* test with Bonferroni’s correction, *P < 0.0167. Error bars in **G**–**I** indicate SEM.

## Version 1 11/25/2025

In-Press Preview

## Version 2 01/16/2026

Electronic publication

## Version 3 03/12/2026

Acknowledgments section added
