# Supplementary material for: Functional interrogation of contextually correct MYH7 variants using CRaTER-flox gene editing and contractility profiling
Source: J Clin Invest. 2025 Nov 25;136(2):e192057. doi: 10.1172/JCI192057 (PMC12807464; doi:10.1172/JCI192057)
Supplement: Supplemental data [file jci-136-192057-s002.pdf]

Supplemental Material

Functional interrogation of contextually-correct *MYH7* variants using CRaTER-flox gene editing and contractility profiling

Alexander M. Loiben[1], Wei-Ming Chien[1], Ashley McKinstry[1], Dania Ahmed[1], Matthew C. Childers[2], Michael Regnier[2], Charles E. Murry[3], and Kai-Chun Yang[1,4]

[1]Department of Medicine/Cardiology, University of Washington, Seattle, WA. [2]Department of Bioengineering, University of Washington, Seattle, WA. [3]Department of Stem Cell Biology and Regenerative Medicine, University of Southern California, Los Angeles, CA. [4]Cardiology/Hospital Specialty Medicine, VA Puget Sound Healthcare System, Seattle, WA.

<u>Correspondence</u>: Kai-Chun (Daniel) Yang; kcyang@uw.edu; 850 Republican St, Seattle, WA 98109; Phone: 206-221-5061

Conflicts of Interest

CEM is a scientific founder and equity holder in StemCardia and equity holder in Sana Biotechnology.

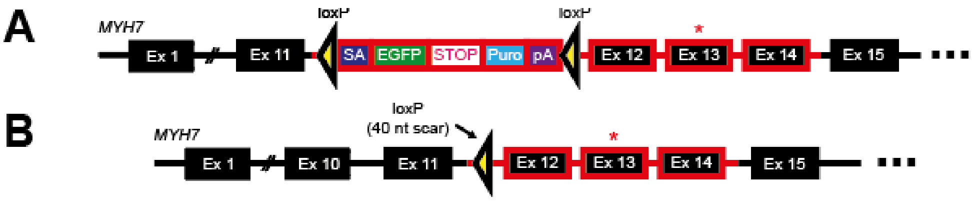

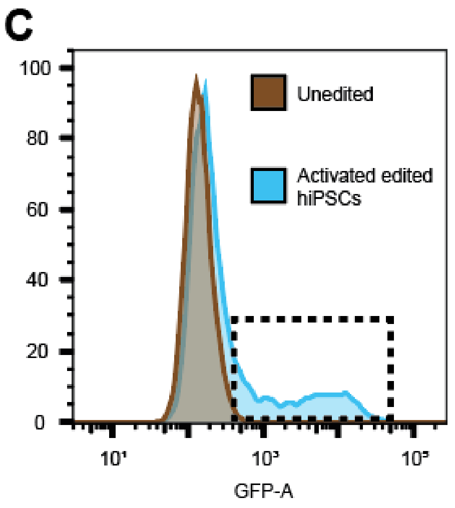

D

hiPSC line	LoxP intronic scar	# Correct	# Total	% Correct
WT	11	48	53	91
WT	14	5	13	38
R369Q	11	7	8	88
P402L	11	7	8	88
R403L	11	6	8	75
R403P	14	4	6	67
R403W	14	5	6	83
R434K	11	1	1	100
T441M	14	6	8	75
R442H	14	3	8	38
R442L	11	15	16	94
R442P	11	6	8	75
Q451K	14	6	7	86
R453C	11	7	8	88
R453H	14	7	8	88
R453R	14	3	8	38
I457M	14	4	8	50
I457T	14	6	8	75
	All colonies	146	190	76.8
			Average per hiPSC line	74.1

E

CRaTER-flox	CRaTER (cDNA knock-in)
40 nt loxP scar in intron	Loss of introns and 3' UTR
Adaptable to any gene and exons	cDNA is limited by size of plasmid repair template
Does not require fluorescent tag in final line	Final line requires fluorescent tag for FACS enrichment

Figure S1: CRaTER-flox editing enables efficient near-scarless generation of variant hiPSCs. (**A**) Schematic of *MYH7* with donor DNA in red knocked in. (**B**) Schematic of edited *MYH7* allele after Cre recombination. (**C**) FACS enrichment of EGFP$^+$ hiPSCs after transient CRISPR activation of *MYH7*. Brown: unedited WTC11 *CLYBL*$^{dCas9\text{-}VPR/dCas9\text{-}VPR}$ *MYH7*$^{WT/WT}$ hiPSCs; blue: WTC11 *CLYBL*$^{dCas9\text{-}VPR/dCas9\text{-}VPR}$ *MYH7*$^{EGFP\text{-}R403W/WT}$ hiPSCs prior to Cre recombination. Boxed region: EGFP$^+$ hiPSC population with (A) genotype; sorted for subsequent Cre recombination. (**D**) CRaTER-flox editing efficiency for generation of variant hiPSC lines. (**E**) Comparison between CRaTER-flox and CRaTER cDNA knock-in gene-editing strategies.

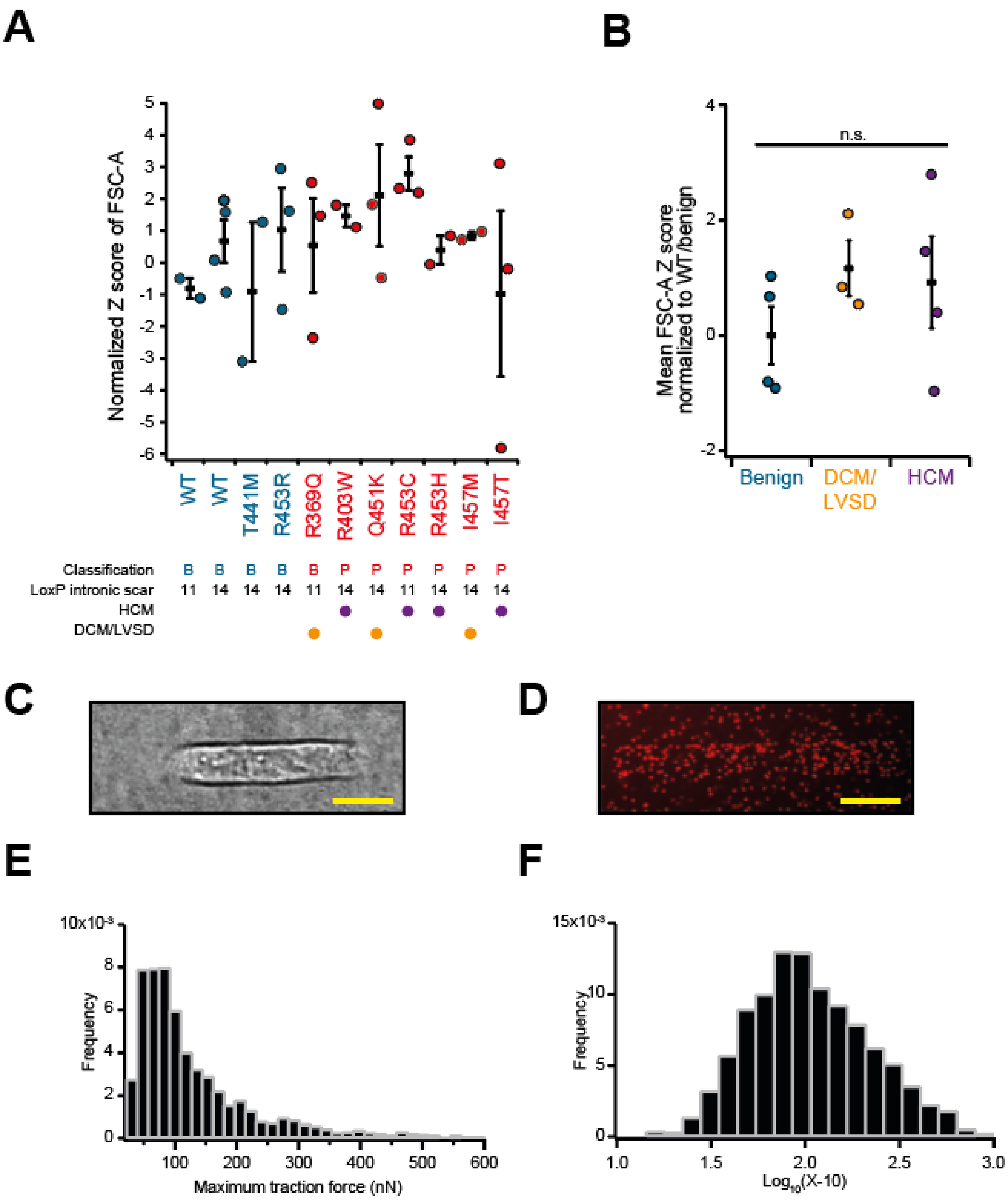

Figure S2: Phenotypic assays to interrogate *MYH7* variant effect in hiPSC-CMs. (**A**) FSC-A Z scores normalized to distribution of control means, n = 2-4 biological replicates per

line, n ≥ 10,000 cells per replicate. Blue: benign/likely benign (B); red: pathogenic/likely pathogenic (P). Circle: variant-associated cardiomyopathy as reported in ClinVar or (4). (**B**) Mean FSC-A Z scores for benign, DCM / LV systolic dysfunction (LVSD), and HCM lines; n.s.: not significant Kruskal-Wallis test at $\alpha = 0.05$. (**C**) Representative brightfield image of hiPSC-CM on patterned hydrogel, 40x objective, scale bar 25 µm. (**D**) Confocal microscopy image of 500 nm red fluorescent beads 5 µm below hydrogel surface corresponding with (E), 40x objective, scale bar 25 µm. (**E**) Combined histogram of maximum traction force values for all tested *MYH7* exon 12-14 lines, n = 1534 cells. (**F**) Combined histogram of maximum traction force values for all tested *MYH7* exon 12-14 lines after $\log_{10}(X-10)$ transformation to convert to a normal distribution, n = 1534 cells.

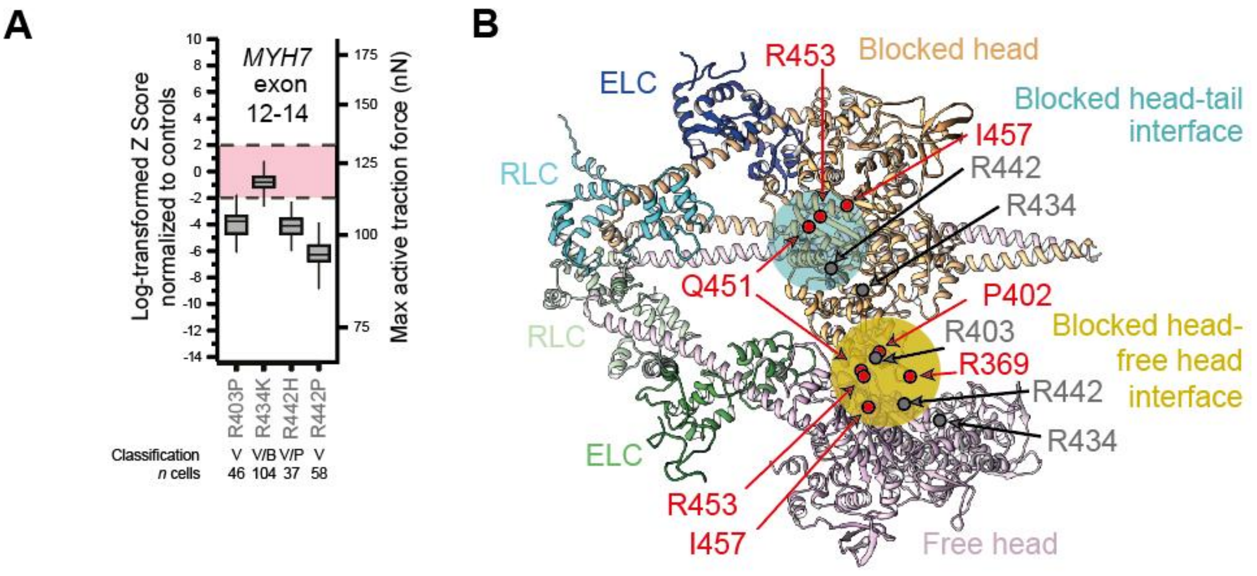

Figure S3: Functional assessment and structural contextualization of *MYH7* VUS. (**A**) Mechanical assessment of *MYH7* VUS in CRaTER-flox edited hiPSC-derived cardiomyocytes. Log-transformed maximum hiPSC-CM active traction force Z scores normalized to benign lines and raw force values. Box: upper and lower quartiles; midline: median; whiskers: 1.5x IQR. V: VUS; B: likely benign; P: likely pathogenic. R434K has conflicting assertions (VUS, likely benign); R442H has conflicting assertions (VUS, likely pathogenic). **(**B) Ribbon structure of a two-headed myosin with essential light chain (ELC) and regulatory light chain (RLC) in the interacting heads motif (IHM) conformation (Protein Database: 8ACT). Gray: VUS residues; red: pathogenic residues.

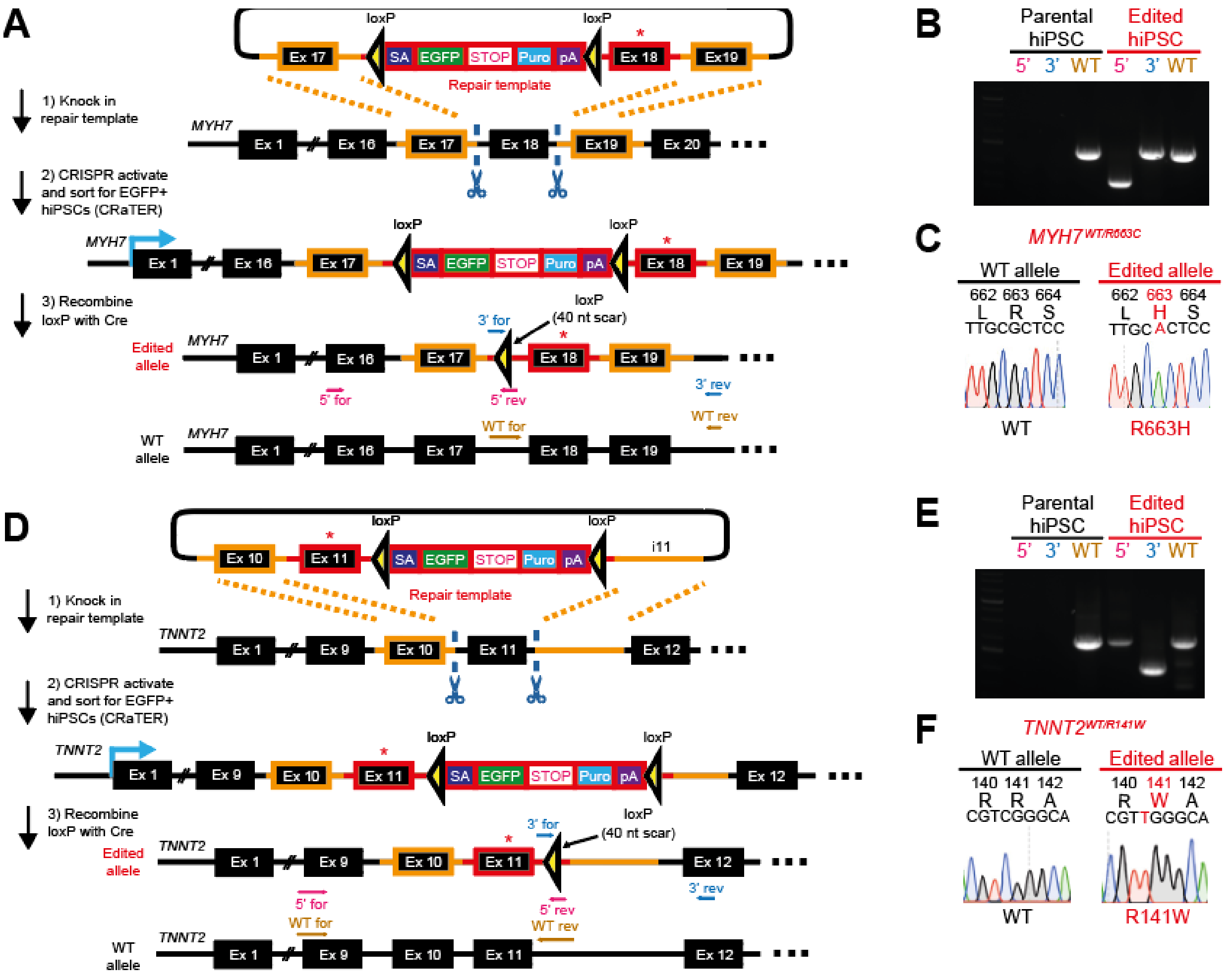

G

MYH7

hiPSC line	LoxP intronic scar	# Correct	# Total	% Correct
WT	17	19	22	86
L655M	17	5	6	83
R663C	17	3	4	75
R663H	17	1	2	50
	All colonies	28	34	82.4
	Average per hiPSC line			73.7

H

TNNT2

hiPSC line	LoxP intronic scar	# Correct	# Total	% Correct
R141R	11	14	14	100
R151R	11	11	12	92
R141W	11	22	24	92
R144G	11	15	18	83
	All colonies	62	68	91.2
	Average per hiPSC line			91.7

Figure S4: CRaTER-flox gene editing of *MYH7* and *TNNT2*. (**A-C**) *MYH7* exon 18 editing strategy. (A) Editing schematic. *variant. (B) Representative genotyping gel. Parental: WTC11 $MYH7^{WT/WT}$; edited: $MYH7^{WT/R663H}$. (C) Representative chromatograms. (**D-F**) *TNNT2* exon 11 editing strategy. (D) Editing schematic. (E) Representative genotyping gel. Parental: WTC11 $TNNT2^{WT/WT}$; edited: $TNNT2^{WT/R141W}$. (F) Representative

chromatogram. (**G-H**) CRaTER-flox editing efficiency for generation of *MYH7* exon 18 (G) and *TNNT2* exon 11 (H) variant hiPSC lines.

Methods

Sex as a biological variable: Our study exclusively examined variants in the context of a human male induced pluripotent stem cell line to ensure an isogenic background. A male cell line was chosen because HCM variants are known to be more penetrant with earlier onset in males compared to females.

hiPSC culture: hiPSCs were maintained on Matrigel-coated plastic using mTeSR Plus media (STEMCELL Technologies) with the addition of Y-27632 rho kinase (ROCK) inhibitor in the first 24h post-replating.

Generation of hiPSC line for heterozygous editing of *MYH7* intron 11-14 ("landing pad"): WTC11 $\textit{CLYBL}^{\text{dCas9-VPR-mCherry/dCas9-VPR-mCherry}}$ hiPSCs were previously generated as described in (3). To remove the mCherry fluorophore so that a red fluorophore could be used in the future if desired, Cas9 protein and three sgRNAs targeting mCherry (UCUGGGUGCCCUCGUAGGGG, GUCGGCGGGGUGCUUCACGU, CUGCAUUACGGGGCCGUCGG) were electroporated with the Neon Electroporation System (1400V, 20 ms pulse, one pulse) into WTC11 $\textit{CLYBL}^{\text{dCas9-VPR-mCherry/dCas9-VPR-mCherry}}$ hiPSCs to delete 284 bp from the mCherry sequence. This yielded WTC11 $\textit{CLYBL}^{\text{dCas9-VPR/dCas9-VPR}}$ hiPSCs.

To create a heterozygous hiPSC "landing pad" line for *MYH7* introns 11-14 that ensures only one *MYH7* allele can be edited, we leveraged CRaTER-flox to first destroy the i11 and i14 gRNA sites on one *MYH7* allele. To achieve this, we created a plasmid repair template containing a lox257-flanked cassette that includes a splice acceptor, EGFP, stop codon, phosphoglycerate kinase 1 (PGK1) promoter, puromycin resistance,

and polyA, analogous to the Repair Template in Figure A but flanked with lox257. The lox257-flanked cassette was then followed by genomic DNA consisting of *MYH7* intron 11 to 14 and a 5-nucleotide deletion in intron 14 that destroys the i14 gRNA site. The plasmid repair template, Cas9 protein, and sgRNAs targeting *MYH7* intron 11 and 14 (CCAAGAAACAAGCATCACCG, TATTTGCTTAGAGCCAGCTG) were electroporated with the Neon Electroporation System (1400V, 20 ms pulse, one pulse) into WTC11 $CLYBL^{dCas9\text{-}VPR/dCas9\text{-}VPR}$ hiPSCs. After puromycin selection, cells were transfected with three sgRNAs targeting 150 bp upstream of the *MYH7* transcriptional start site (TCACTAGAAGCATTTCCCCC, GTCCCTAGCCGGATTTAGAAA, GTTATGGCATGGACTGTGCA) and FACS enriched for EGFP expression after 48-72h. Enriched cells were treated with TAT-Cre recombinase (1 µM, Excellgen #EG-1001) for 24h to flox out the EGFP and puromycin selection cassette. Single colonies were picked and screened for the desired heterozygous genotype (one WT allele and one 5-nucleotide deletion in intron 14 that destroys the i14 gRNA site), establishing a heterozygous "landing pad" line that destroys the gRNAs sites for i11 and i14 on one *MYH7* allele (the recombined loxP257 site destroys the i11 gRNA site). This ensures that subsequent CRaTER-flox editing of *MYH7* introns 11-14 will only occur on one allele. Lox257 was selected for the generation of the landing pad line so that it would not recombine with wildtype loxP site, which is used for the CRaTER-flox gene editing of the variant allele.

<u>CRaTER-flox gene editing</u>: *MYH7* exon 12-14 variants with the intron 11 scar used the "landing pad" hiPSC line (which ensures heterozygous gene editing), while all other variant hiPSC lines (*MYH7* exon 12-14 variants with the intron 14 scar, *MYH7* exon 18 variants, *TNNT2* exon 11 variants) used the parental WTC11 $CLYBL^{dCas9\text{-}VPR/dCas9\text{-}VPR}$

hiPSC line. All gene edited lines that started with the parental WTC11 $\mathit{CLYBL}^{dCas9\text{-}VPR/dCas9\text{-}VPR}$ hiPSC line were screened to ensure the presence of one variant allele and one WT allele (as demonstrated in Figure B, Figure S4 B, E). For CRaTER-flox gene editing, the parental hiPSC line was electroporated with an appropriate plasmid repair template (Figure A, Figure S4 A, D), Cas9, and appropriate sgRNAs (*MYH7* Exon 18: CCTGTCTCCTTGGTGCATTC, GATGGACATTACCTCATCAG; *TNNT2*: TACCCTGGTGTGCACGTCCG, GGAGCATGGGGGCCTCCAT) using the same electroporation settings as above. After puromycin selection, cells were electroporated with sgRNAs upstream of the *MYH7* or *TNNT2* transcriptional start site (*TNNT2*: CACATGGGCTTATATGGCGT, TGTTCCTGTAGCCTTGTCCC) and FACS-enriched for EGFP expression after 48-72h. Enriched cells were treated with TAT-Cre recombinase for 24h, yielding a 40-nucleotide intronic scar (34-nucleotide LoxP sequence and 6-nucleotide EcoRV restriction enzyme cloning artifact). Single colonies were picked and screened for the correct genotype, establishing isogenic clonal variant hiPSC lines.

<u>hiPSC-CM differentiation</u>: hiPSCs were differentiated using established protocols via timed activation and inhibition of Wnt in L-ascorbic acid, bovine serum albumin and RPMI media, then maintained with RPMI cardiomyocyte media with B-27 Plus supplement (Gibco). On differentiation day 20, hiPSC-CMs were metabolically purified in media supplemented with lactate yet deprived of glucose or pyruvate for 5d. At differentiation day 25, hiPSC-CMs were cryopreserved in Cryostor CS10 (StemCell Technologies, #07-930) at $4x10^6$ cells/mL density. hiPSC-CMs were thawed at 250k cells/cm^2 on Matrigel coated plastic and fed with DMEM cardiomyocyte media for recovery from cryopreservation prior to seeding on differentiation day 30.

Forward scattering area (FSC-A): Differentiation day 30 hiPSC-CMs were seeded at 100k cells/cm^2 on Matrigel coated plastic and fed with DMEM cardiomyocyte media for 10 ± 2d. Cells were lifted and FSC-A was measured for single cells using flow cytometry with consistent voltage. 10,000 cells per biological replicate was collected.

Traction force microscopy (TFM): TFM polyacrylamide hydrogels were created using established protocols (5). Briefly, polydimethylsiloxane (PDMS) stamps were generated using silicon wafer patterns with 17x119x10 µm rectangles (Research Microstamps) and coated overnight with 1 mg/mL Matrigel. Patterns were imprinted onto 25 mm glass coverslips. Polyacrylamide hydrogels of physiological stiffness (10 kPa) were created with 500 nm diameter red fluorescent microbeads (Thermo Fisher, F8812) uniformly embedded. After post-cryopreservation recovery, differentiation day 30 hiPSC-CMs were seeded (100k cells, 250 µl) for 1h and then fed with DMEM cardiomyocyte media for 14 ± 7d. Cells were electrically stimulated at 1 Hz in 1x Tyrode's solution and fluorescent beads 5 µm below the hydrogel surface were imaged for 6s at 15 fps with 40x spinning disk confocal objective. Bead displacements and corresponding raw traction forces were calculated using previously published Matlab software (5).

Statistics: For FSC, maximum contraction velocity, and maximum relaxation velocity, raw values were converted to Z scores based on the distribution of mean values for control lines (N = 4-6). Mean values for benign, DCM, and HCM lines were compared by Kruskal-Wallis test with $p < 0.05$ considered significant, and then post hoc confirmed with 2-tailed Mann-Whitney U test with $p < 0.0167$ considered significant after Bonferroni correction for 3 pairwise comparisons. For maximum traction force, raw values for each cell were transformed into a normal distribution using the formula $Y = \log10(X-10)$, where X is the

raw value and Y is the transformed value. The transformed distribution was not significantly different from a normal distribution based on D'Agostino-Pearson normality test for skewness and kurtosis, with $p < 0.05$ considered significant. Transformed values were converted to Z scores based on the distribution of mean values for the benign variants across all editing schemes (N = 8). Mean Z scores for benign, DCM, and HCM lines were compared by Kruskal-Wallis test with $p < 0.05$ considered significant, and then post hoc confirmed with 2-tailed Mann-Whitney U test with $p < 0.0167$ considered significant after Bonferroni correction for 3 pairwise comparisons.

Data availability: Values for all data points in graphs are reported in the Supporting Data Values file. TFM videos and other raw data files are available from the Authors upon request.

Author contributions*:* A.M. Loiben designed research experiments, conducted experiments, analyzed data, wrote the manuscript, and edited the manuscript. W-M. Chien designed research experiments, conducted experiments, analyzed data, and edited the manuscript. A. McKinstry conducted experiments and analyzed data. D. Ahmed conducted experiments and analyzed data. M.C. Childers analyzed data and edited the manuscript. M. Regnier edited the manuscript, provided supervision, and acquired funding. C.E. Murry edited the manuscript, provided supervision, and acquired funding. K-C. Yang designed the research experiments, wrote the manuscript, edited the manuscript, provided supervision, administered the project, and acquired funding.

Acknowledgements: The authors would like to acknowledge the support of Dale Hailey, Director, Lynn and Mike Garvey Imaging Core, Institute for Stem Cell and Regenerative

Medicine, University of Washington, with assistance with microscopy. The authors would also like to acknowledge Chuck Maynard, Research Professor Emeritus, Health Systems and Population Health, University of Washington, for assistance with statistical analysis.

Funding sources: NIH F32HL164108 (A.L.), NIH K99HL173646 (M.C.), NIH P30AR074990 (M.R.), NIH R01HL128368 (M.R.), NIH R01HL160825 (C.M.), NIH R01HL148081 (C.M.), R01HL146868 (C.M.), Robert B. McMillen Foundation (C.M.), NIH R01HL171174 (K-C.Y.), VA I01BX006428 (K-C.Y.), VA IK2BX004642 (K-C.Y.), John L. Locke, Jr. Charitable Trust (K-C.Y.).

Disclaimer: The content is solely the responsibility of the authors and does not necessarily represent the official views of the Department of Veterans Affairs or the US Government.
