# Supplementary material for: Functional interrogation of contextually correct MYH7 variants using CRaTER-flox gene editing and contractility profiling
Source: J Clin Invest. 2025 Nov 25;136(2):e192057. doi: 10.1172/JCI192057 (PMC12807464; doi:10.1172/JCI192057)

Full unedited gel for Figure B

Lanes used in Figure B

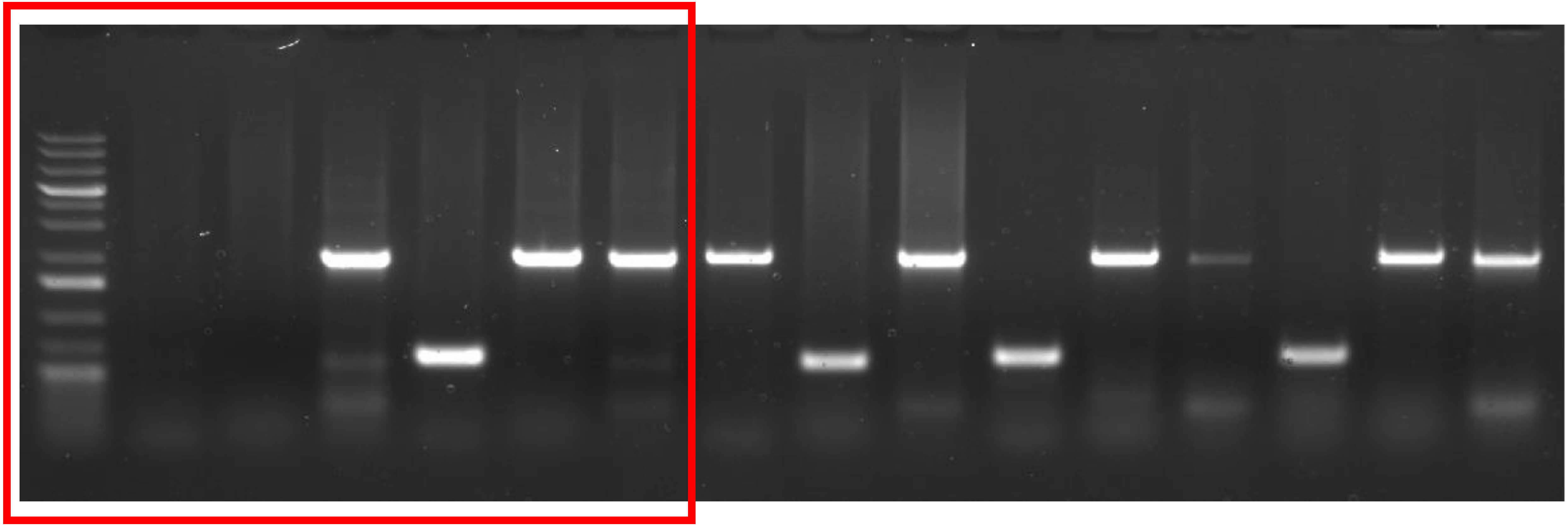

Full unedited gel for Figure 4B

Lanes used in Figure 4B

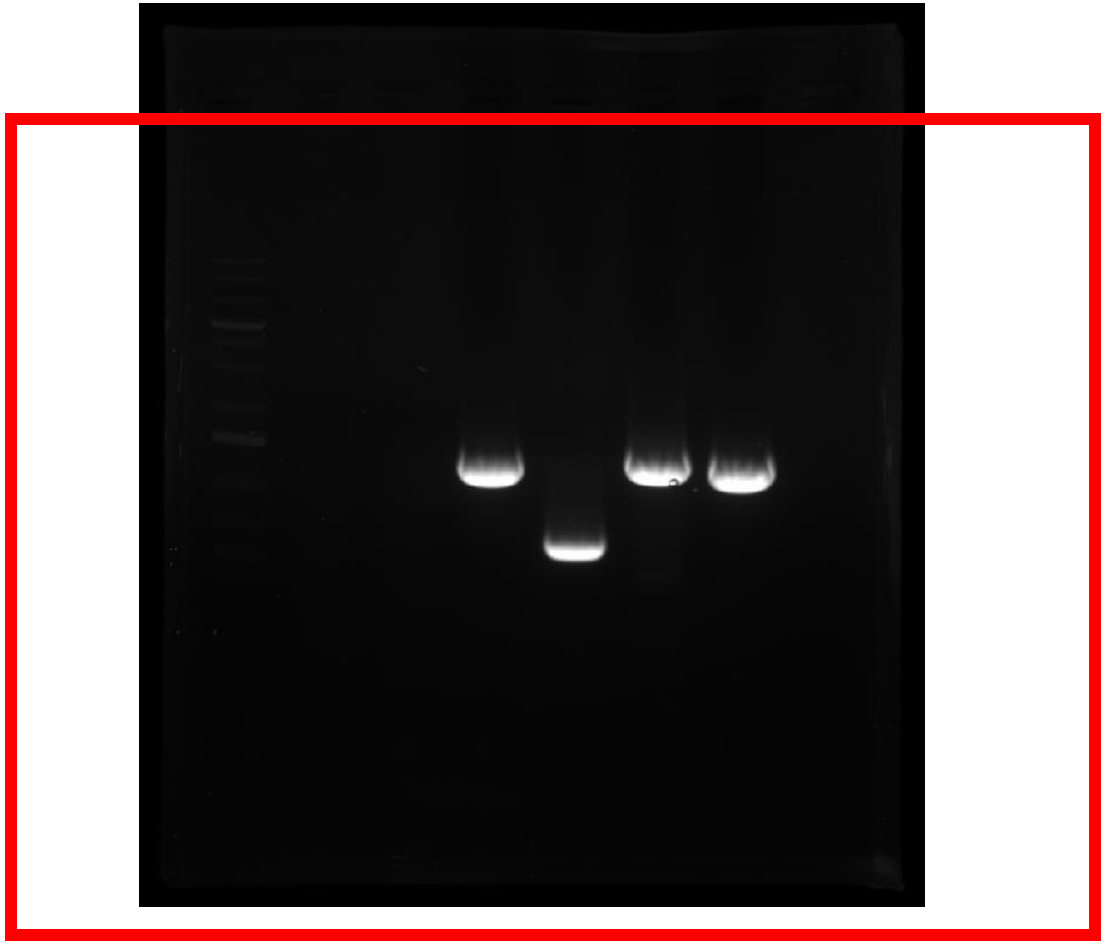

Full unedited gel for Figure 4E

Lanes used in Figure 4E

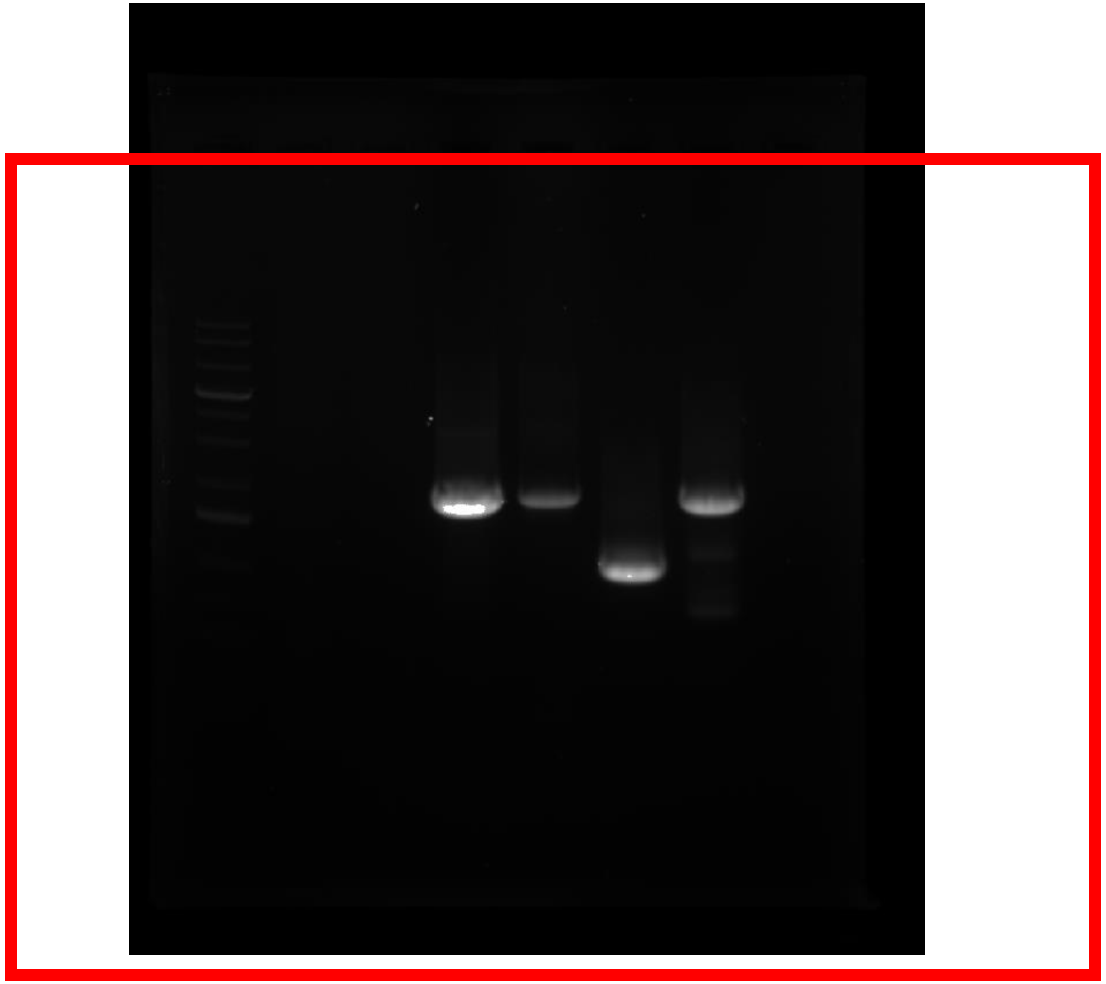

Supplement: Unedited blot and gel images [file jci-136-192057-s003.pdf]
